# Periodontitis: A Multifaceted Disease of Tooth-Supporting Tissues

**DOI:** 10.3390/jcm8081135

**Published:** 2019-07-31

**Authors:** Eija Könönen, Mervi Gursoy, Ulvi Kahraman Gursoy

**Affiliations:** 1 Department of Periodontology, Institute of Dentistry, University of Turku, 20520 Turku, Finland; 2 Oral Health Care, Welfare Division, City of Turku, 20101 Turku, Finland

**Keywords:** periodontal disease, alveolar bone loss, gingiva, bacteria, biofilm, immunity, inflammation, smoking

## Abstract

Periodontitis is an infection-driven inflammatory disease in which the composition of biofilms plays a significant role. Dental plaque accumulation at the gingival margin initiates an inflammatory response that, in turn, causes microbial alterations and may lead to drastic consequences in the periodontium of susceptible individuals. Chronic inflammation affects the gingiva and can proceed to periodontitis, which characteristically results in irreversible loss of attachment and alveolar bone. Periodontitis appears typically in adult-aged populations, but young individuals can also experience it and its harmful outcome. Advanced disease is the major cause of tooth loss in adults. In addition, periodontitis is associated with many chronic diseases and conditions affecting general health.

## 1. Introduction

**Figure 1:**
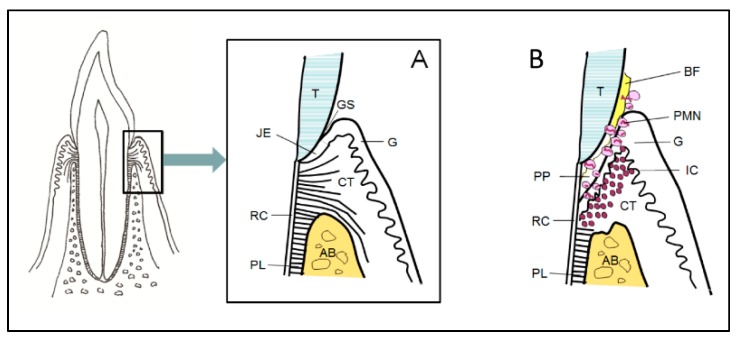
The anatomical structure of the periodontium in health (**A**) and in periodontitis (**B**). Abbreviations: Alveolar bone (AB), bacterial biofilm (BF), connective tissue (CT), gingiva (G), gingival sulcus (GS), inflammatory cells (IC), junctional epithelium (JE), polymorphonuclear neutrophils (PMN), periodontal ligament (PL), periodontal pocket (PP), root cementum (RC), and tooth (T).

Periodontitis is an infection-driven inflammatory disease in tooth-supporting tissues (i.e., the periodontium). Moreover, genetics and environmental and behavioral factors are involved in the development of the disease, the exposure of susceptible individuals to its initiation, and the speed of progression. The structure of the periodontium is diverse; it is composed of the gingiva, the underlying connective tissue, cement on the root surface, alveolar bone, and the periodontal ligament between the cementum and alveolar bone (Figure 1A,B). The junctional epithelium of the gingiva is a unique structure, located at the bottom of the gingival sulcus, which controls the constant presence of bacteria at this site. The most characteristic feature of periodontitis is the activation of osteoclastogenesis and the destruction of alveolar bone as its consequence, which is irreversible and leads to loss of tooth support.

Periodontal disease, especially its mild and moderate forms, is highly prevalent in adult-aged populations all over the world, with prevalence rates around 50% [1], while its severe form increases especially between the third and fourth decades of life, with the global prevalence being around 10% [2]. Certain demographic characteristics, such as age, gender, ethnicity, and socioeconomic status, influence the prevalence of periodontitis. Other strongly contributing factors include smoking, diabetes mellitus, metabolic syndrome, and obesity [3,4]. It is noteworthy that smoking and diabetes can expose individuals to the advanced form of periodontal disease already in adolescence and early adulthood [5,6,7]. There is also a strong relation of smoking to tooth loss in young individuals [8]. Severe periodontitis, the major cause of tooth loss in adults (https://www.nidcr.nih.gov/research/data-statistics/periodontal-disease), is typically complicated by the drifting and hypermobility of teeth, eventually resulting in the collapsed bite function of an affected individual [9,10]. Moreover, periodontal disease as well as tooth loss are considered to have an association with a variety of chronic diseases and conditions affecting general health.

Even in periodontal health, immune cells are constantly present in the gingiva, thus supporting the balance between oral biofilms and the host [11]. This constant communication keeps the immune response active, being a reciprocal, synergistic, and dynamic interaction. In the periodontium, the immune response carries characteristics of that of any other part of the body; the first action against microbes is due to non-specific innate response, while extended pathogenic challenge activates specific adaptive responses.

Excessive dental plaque accumulation at the gingival margin leads to inflammation and increasing proportions of proteolytic and often obligately anaerobic species [12]. The presence of periodontal species with pathogenic potential in the gingival sulcus initiates an inflammatory response in gingival tissue. When allowed to become chronic, this can have drastic consequences in the periodontium of susceptible individuals. Interactions between the components and metabolic activities of the oral microbiota and the host either support the balance (homeostasis) or result in disturbance (dysbiosis) within the microbiota [12]. Periodontal health-associated commensals are important in protecting the balance, for example, by inhibiting the growth of periodontitis-associated pathogens. However, qualitative and quantitative alterations within subgingival biofilms can result in disrupted homeostasis, which consequently can lead to the onset of disease with various degrees of periodontal tissue destruction.

## 2. Pathogenic Biofilms

Multispecies biofilm formation and maturation occur on tooth surfaces via intergeneric interactions, where coaggregations occur between different bacterial taxa, and highly diverse bacterial communities are formed at supragingival (above the gumline) and subgingival (below the gumline) sites [13,14]. *Fusobacterium nucleatum*, which belongs to the core anaerobic microbiota of the oral cavity from early years of life onwards [15,16], is seen as an important bridging organism of maturing dental biofilms, allowing late-colonizing species with virulent properties to be colonized [13]. This gradual maturation and shifts in the microbial composition influence the pathogenicity of subgingival biofilms where metabolically highly specialized microorganisms function in physical proximity as interactive microbial communities [12]. Focusing on only its adherence capabilities may lead to the underestimation of other important virulence characteristics of *F. nucleatum*, a relevant bacterium in the initiation and progression of periodontal disease. In a biofilm, this obligate anaerobe can survive and increase its numbers in aerobic environments [17]. Indeed, recent evidence indicates that *F. nucleatum* induces an environmental change through hypoxia [18], which can support the colonization of anaerobic pathogens in dental biofilms. The effects of *F. nucleatum*-induced hypoxia are not limited to the shifts in the biofilm composition—this hypoxia also directs endothelial cells to an inflammatory state and activates angiogenesis [18].

In subgingival biofilms, anaerobic gram-negative species with their biologically active lipopolysaccharide (LPS)-containing cell wall structure may be essential in awakening the inflammatory reaction in the gingiva and culprits of periodontal destruction to occur in periodontitis-susceptible individuals [11]. Subgingival plaque samples collected from periodontitis patients and periodontitis-free individuals differ from each other. *Porphyromonas gingivalis*, *Tannerella forsythia*, and *Treponema denticola*, the so-called red complex, have shown the strongest association with periodontal disease [19]. In another study by using 454 pyrosequencing of 16S rRNA genes, *P. gingivalis* and *T. denticola* but also *Filifactor alocis*, a gram-positive anaerobe, formed the top three species [20]. Of these, *P. gingivalis* is suggested to be the principal pathogen in the process, causing a disturbed interplay between the subgingival biofilm and the host response [21]. Even as a minor constituent of the subgingival microbiota, it is able to severely affect the ecosystem by influencing the numbers and community organization of commensal bacteria at the site and dysregulate innate immunity pathways. *P. gingivalis*, a highly proteolytic gram-negative anaerobe, is a common recovery from deepened periodontal pockets of adult periodontitis patients. While *P. gingivalis* is rather rare in children and adolescents, its salivary carriage rates increase significantly with aging, and it is detected in the majority of the Finnish population after the age of 55 years [22]. Different from *P. gingivalis*, the carriage of *Aggregatibacter actinomycetemcomitans* was less frequent, without any connection to age. This gram-negative capnophilic coccobacillus and an established periodontal pathogen has been linked to aggressive forms of periodontal disease [23]. Besides these traditional pathogens, open-ended molecular methods have significantly enlarged the list of pathogenic species within periodontitis-associated subgingival biofilms [24].

Many periodontitis-associated species, among those *A. actinomycetemcomitans*, *P. gingivalis*, and *F. nucleatum*, or even polymicrobial aggregates, are capable of invading periodontal tissues [25,26,27], thus evading many defense mechanisms of the host. This, in turn, has an impact on the persistence of inflammation and progression of periodontal tissue destruction.

For periodontal disease to occur, it is not the presence of a single periodontal pathogen, but the interplay between the composition of the subgingival biofilm and the host response where host factors and specific niches play an important role. In dysbiotic biofilms, there is abundance of immunostimulatory pathobionts and their virulence factors, but also a reduced inhibitory effect of commensal bacteria, thus resulting in an increased inflammatory response [28,29]. In gingival epithelia, cellular responses are especially elicited against polymicrobial biofilms due to their interbacterial metabolic and virulence synergisms [30], leading the way to initial pocket formation and attachment loss. Deepening periodontal pockets with an anaerobic environment, inflammatory conditions, and a large amount of substrates originating from tissue destruction all favor the growth of inflammophilic periodontal pathogens and pathobionts [21]. Notably, daily smoking contributes to further disturbances in the subgingival microbiota, facilitating an abundance of periodontal pathogens and the reduction of beneficial commensals, thus exposing smokers to periodontal disease [31].

There have been intensive research activities targeting periodontitis-associated bacteria and/or the antibodies working against them, with the aim of revealing their involvement in various systemic diseases and conditions. Major periodontal pathogens raise local and systemic antibody responses; it has been shown in multivariate analyses that the main determinant of the systemic antibody response to *P. gingivalis* and *A. actinomycetemcomitans* is the carriage of the pathogen, whereas the presence or degree of periodontal disease has only a modest modifying effect [32]. High serum IgG antibodies to major periodontal pathogens act as risk factors for future cardiovascular events [33,34,35]. Also, the circulation of LPS of virulent gram-negative pathogens (endotoxemia) accompanied by exaggerated proinflammatory responses is connected to risk for these events [33]. Subgingival bacteria, including *P. gingivalis* and *A. actinomycetemcomitans*, have also been associated with the prevalence of prediabetes in young diabetes-free adults [36]. Interestingly, associations between bacterial measures and prediabetes are consistently stronger than those between periodontitis and prediabetes. Furthermore, there is accumulating evidence on the role of *P. gingivalis* in rheumatoid arthritis [37,38]. A prospective study on the association between pancreatic cancer and the oral microbiome revealed that the carriage of *P. gingivalis* and *A. actinomycetemcomitans* is a subsequent risk for this highly lethal cancer type [39]. Besides the impact of *F. nucleatum* as the key organism in dental biofilms and its involvement in oral and extra-oral polymicrobial infections [40], recent research has shown its significant carcinogenic potential. Its ability to tolerate oxygen, create hypoxia, and induce an inflammatory environment may explain the role of *F. nucleatum* in the development and progression of colorectal adenocarcinoma [41].

## 3. Immunologic Players of the Periodontium

Due to the constant interaction with bacteria, immune cells (neutrophils, macrophages, and lymphocytes) are present in the periodontium to take part in maintaining a healthy equilibrium. Neutrophils continuously transmigrate through the junctional epithelium to gingival sulcus and release antimicrobial peptides (α-defensins) against invading bacteria, while they also stimulate adhesion and the spread of keratinocytes on the tooth surface [42]. Resident cells of the periodontium (keratinocytes, fibroblasts, dendritic cells, and osteoblasts) are not passive barriers against bacterial invasion, but they initiate innate immune response and regulate adaptive immune response [43,44]. An essential component is the complement pathway, which activates, amplifies, and synchronizes innate immune response by opsonizing and killing bacteria as well as activating mast cells, neutrophils, and macrophages of the periodontium [45].

**Figure 2:**
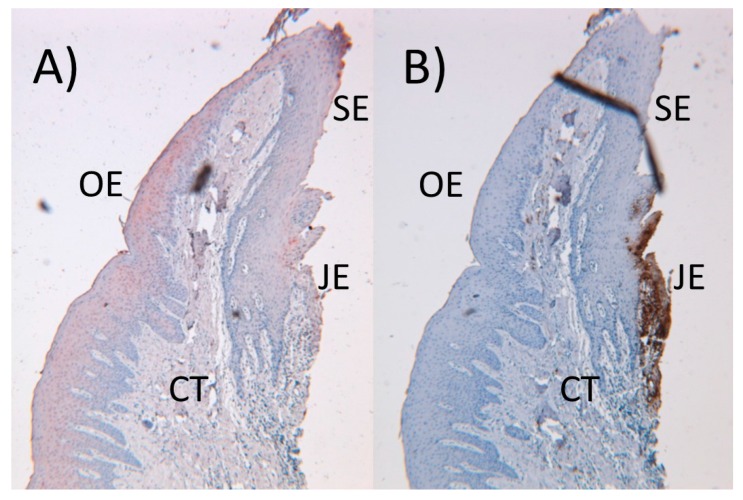
In a healthy gingiva, epithelial defensins (human β-defensins (hBD-2) in red color) are located in the oral (OE) and sulcular (SE) epithelia (**A**), while neutrophilic antimicrobial peptides (α-defensins in brown color) are located in the junctional epithelium (JE) and partly in connective tissue (CT) (**B**). (An original figure by U.K.G.)

Keratinocytes, which form the majority of the gingival epithelium, are capable of producing and secreting various immune response mediators, among them human β-defensins (hBDs), cathelicidins, proinflammatory cytokines, chemokines, and angiogenetic proteins [46,47]. In the healthy gingiva, innate response is mainly regulated by keratinocytes and neutrophils; keratinocytes secrete hBDs to protect the oral and sulcular epithelium (Figure 2A), whereas neutrophils secrete α-defensins to protect the junctional epithelium (Figure 2B). Gingival keratinocytes recognize pathogen-associated molecular patterns (PAMPs) by their pattern recognition receptors, such as toll-like receptors (TLRs). mRNA expressions of TLR 1–9 are detected in connective tissue and epithelial layers of the gingiva [48]. In addition, bacterial signaling molecules (cyclic dinucleotides and quorum signaling molecules) activate cytokine response in gingival keratinocytes [49,50]. There is also a reciprocal interaction between innate-immune proteins and keratinocytes. For example, proinflammatory interleukins (IL-1α, IL-1β, IL-6) activate the protein expression and secretion of hBDs from keratinocytes [46,51], while keratinocytes can suppress the inflammatory response by secreting monocyte chemotactic protein-induced protein-1 [52].

Gingival connective tissue, periodontal ligament, and the organic component of the bone are formed of collagen. Fibroblasts are responsible for the synthesis of new collagen bundles and they remove the old collagen by secreting matrix metalloproteinases (MMPs). Overexpression of MMPs by gingival fibroblasts may either induce the release of cytokines and chemokines from the extracellular matrix or cleave cytokines and interrupt immune response signaling cascades [53]. The interplay between neutrophils and gingival fibroblasts is a good example of the bidirectional interactions between resident and immune cells.

Dendritic cells differ from keratinocytes and fibroblasts by acting as phagocytes and antigen-presenting cells. In a healthy environment, dendritic cells are in their immature forms and have high phagocytic capacity against invading microorganisms, but during infection they initiate a maturation process that involves their migration to lymph nodes to activate CD4^+^ T cells [54] and promote the polarization of T-helper (Th)1, Th2, Th17, and B cells [55]. Uncontrolled upregulation of Th1 and Th17 cell pathways enhances alveolar bone loss via the induction of osteoclastogenesis [56]. There is also evidence that dendritic cells can differentiate to osteoclasts [57]; however, it is unknown how much of the bone resorption seen in periodontitis is actually induced by dendritic cell-derived osteoclasts.

Neutrophils form the primary defense system in periodontal tissues. Notably, their migration through the junctional epithelium into the gingival sulcus is a continuous process, which may differ from other organs, where transmigration is a hallmark of infection [58]. In the healthy oral cavity, neutrophil populations tend to be parainflammatory, while proinflammatory neutrophil phenotypes are present in periodontal disease [59]. Severe forms of periodontitis can be connected to diseases with neutrophil function defects, such as leukocyte adhesion deficiency 1 (LAD-1). Lack of neutrophil surveillance against bacterial infection is considered the cause of excessive periodontal degradation in neutrophil function deficiencies. However, recent evidence indicates that the absence of neutrophils in LAD-1 leads to the overproduction of IL-17, which eventually enhances the proliferation and differentiation of B cells [60]. Chronic granulomatous disease (CGD) is another genetic disease, which is characterized by defective neutrophilic respiratory burst and bacterial elimination. Although CGD patients are prone to developing bacterial and fungal infections, their periodontitis prevalence does not differ from that of the general population. In the highlights of these two examples, the presence of neutrophils in the periodontal immune response cascade seems to be more important than their ability to kill bacteria, as other phagocytes can take care of bacterial killing [60]. Interestingly, peripheral blood neutrophils of periodontitis patients release higher levels of proinflammatory cytokines and reactive oxygen species compared to periodontally healthy individuals, and this hyperinflammatory response persists even after successful periodontal treatment [61,62].

Neutrophils have a relatively short lifespan and they are programmed to die via apoptosis. Apoptotic neutrophils are phagocytosed from tissues by macrophages and are eliminated through lymphatics (efferocytosis). Since neutrophils produce and secrete a significant number of inflammatory molecules, their removal is a hallmark of healing. In inflamed periodontal tissues, partly due to pathogenic biofilms, there is an extended recruitment of neutrophils and delayed apoptotic cell death [63]. Instead of enhanced elimination of pathogens, however, neutrophils demonstrate impaired antibacterial function with this uncontrolled and extended immune response activation [64].

Tissue macrophages derive either from circulating monocytes or from embryo-derived precursors [65]. Phenotyping them as inflammatory and resolving macrophages will define their roles in disease and health. Inflammatory macrophages produce and secrete a large group of cytokines (IL-1β, IL-23, IL-6, tumor necrosis factor (TNF)-α) and enzymes (MMPs) that take part in osteoclastogenesis and collagen degradation in periodontitis [66]. A conversion from a destructive inflammatory phenotype to a resolving and bone-forming phenotype requires both signaling molecules and the presence of apoptotic neutrophils [67]. *P. gingivalis* can reverse the conversion of inflammatory macrophages to resolving macrophages by inducing inflammatory cytokines [68]. Impaired elimination of neutrophils by macrophages and defects in the activation of resolving macrophages may in turn lead to the initiation and progression of periodontitis.

Innate immune cells present intra- and extracellular pathogens to lymphocytes. In the gingiva, the most common subset of lymphocytes is CD4^+^ T cells, followed by CD8^+^ T cells, which are further subgrouped as Th1, Th2, Th17, Th9, regulatory (Treg), and unconventional γδ T cells [69]. Recent evidence indicates the role of Th17 cells as one main regulator of T cell response and bone resorption in the periodontium [70]. In addition, Treg cells can limit the progression of periodontal disease without suppressing the immune response. When chronic gingivitis progresses to periodontitis, there is a shift from T cell dominance to B and plasma cells. Different types of B cells include naive B cells, memory B cells, and antibody-secreting B cells. Antibodies produced against periodontitis-associated pathogens can be found in saliva and in serum as well [32].

Finally, periodontitis is a complex disease with a nonlinear character, and its effects on immune response are rather disproportional [71]. Although knowledge about immune cell functions has considerably increased, it is still difficult to fully understand cellular interactions in periodontal disease pathogenesis due to its multicausal etiology.

## 4. Inflammatory Process and Periodontal Tissue Destruction

The junctional epithelium forms a unique seal between the root surface and gingiva, and its main function is to provide protection to the underlying tissues against the constant exposure of oral microbes and their by-products [72]. Various molecular factors involved in adhesion, cell–cell interactions, chemotaxis, proinflammatory cytokines, epithelial growth, MMP activation, and antimicrobial peptide production contribute to the function of the junctional epithelium. If this elegant and well-adapted defense system is overwhelmed by bacterial virulence factors (e.g., *P. gingivalis* gingipains) and prolonged inflammation (clinically seen as gingival bleeding and changes in soft tissue contour and color), the junctional epithelium migrates apically on the root surface and activates collagen destruction, which eventually leads to periodontal pocket formation [72]. It is noteworthy that although gingival inflammation is the precursor of periodontitis and a clinically relevant risk factor for disease progression, not all gingivitis lesions lead to periodontitis [73]. During periodontal pocket formation, new tissue formation by resident cells (keratinocytes, fibroblasts, osteoblasts) is suppressed, whereas tissue degradation by neutrophils, macrophages, and osteoclasts is stimulated; thus, the balance between tissue removal and regeneration is disrupted [74].

Proinflammatory cytokines (IL-1β, IL-6, IL-23, TNF-α), chemokines (IL-8), and antimicrobial peptides produced by keratinocytes, fibroblasts, and dendritic cells are chemoattractant gradients for neutrophils, which migrate into inflamed tissues and stimulate the chemotaxis of nonresident cells (macrophages, lymphocytes, plasma cells, and mast cells) to the site of infection [43,44]. Phagocytic cells mainly aim to eliminate invading pathogens by producing and secreting antimicrobial agents, reactive oxygen species, and enzymes. However, abundant tissue concentrations of collagenolytic MMPs and elastase activate the degradation of type I collagen in the connective tissue and periodontal ligament [75]. During disease, MMP-8 is the major collagenase in periodontal tissues. Irreversible periodontal destruction occurs when the inflammatory cell infiltrate, predominantly containing plasma cells, extends deeper into the connective tissue, leading to tissue damage in periodontal ligament and alveolar bone [76].

Alveolar bone resorption is the principal pathological characteristic of periodontitis. The activation of osteoclasts, multi-nucleated bone-resorbing cells, is regulated by a cascade of inflammatory proteins (cytokines) and enzymes (MMPs). IL-1β, IL-6, and TNF-α are the major proinflammatory cytokines in osteoclastogenesis activation, which is achieved by upregulating the receptor of nuclear factor-kappa ligand (RANKL) expression and inhibiting the differentiation of osteoblasts as well as decreasing osteocalcin production and new bone formation [77]. Due to the upregulated RANKL (stimulator of mature osteoclast formation) and downregulated osteoprotegerin (blocker of RANKL action), degradation of the bone is enabled to progress. MMP-1, -8, and -13 are especially involved in alveolar bone destruction by degrading type I collagen (the main type of collagen in the periodontium), while two gelatinases (MMP-2 and -9) accomplish the degradation of denatured collagen [78]. Furthermore, MMP-9 assists in osteoclast migration and MMP-13 triggers osteoclast activation, which all facilitate type I collagen degradation.

The disease development with fast or slow progress and with stable periods varies among periodontal sites and among individuals. Diagnosis of periodontitis is based on clinical and radiographic information on periodontal attachment and alveolar bone loss. In the current classification system, staging estimates the severity of the disease, while grading aims to estimate the rate of its progression, taking the known risk factors into account [10]. At the early phase of periodontal disease, the clinical signs and symptoms can be lacking or very mild. When periodontal tissue destruction proceeds, deepened pocket depths with alveolar bone loss result in tooth mobility, drifting, flaring, and finally loss of the affected tooth. In advanced cases, where several teeth are affected, these abnormalities lead to the collapse of the bite function.

## 5. Periodontal Therapy—Impact on Oral and General Health

The primary goal of periodontal therapy is to reduce the infectious and inflammatory challenge and to halt the progressing tissue destruction. Removal of pathogenic biofilms and suppression of inflammation can discontinue the periodontal tissue degradation; however, only limited regain of lost tissues occurs, depending on the form of tissue defects, systemic health status, and age [79]. In advanced cases, the active anti-infective treatment phase is often combined with surgery to eliminate residual pockets—with the aim of improving the ecology at periodontal sites—or sometimes with adjunctive systemic antimicrobials to reduce pathogen burden. In smokers, however, the treatment outcome is compromised, which makes smoking cessation an essential part of their periodontal therapy [80,81]. The beneficial influence of quitting may partly be due to decreased pathogen numbers and increased abundance of health-associated commensals in subgingival biofilms [82]. Although anti-infective treatment reduces total bacterial counts, proportions of periodontal pathogens, as well as the number of sites colonized with pathogens, many of the species return with time [83]. Therefore, daily oral hygiene of the patient and continuing professional supportive periodontal therapy are necessary to maintain the outcome and strengthen the long-term success of the treatment [84,85]. Moreover, patients with advanced disease and masticatory dysfunction and bite collapse due to severe tooth loss have an obvious need for complex rehabilitation of the bite function as well as esthetic treatment. After treatment, however, periodontitis patients with prosthodontic reconstructions have still an increased risk for tooth loss, and many patient-related factors such as age, socioeconomic status, non-compliance, and diabetes are associated with abutment tooth loss [86].

Since untreated periodontitis increases systemic low-grade inflammation, another treatment goal is to improve this condition [87]. Although intensive mechanical periodontal treatment of patients with severely damaged periodontal tissues can cause an acute systemic inflammatory response and impair endothelial function, this occurs only transiently and, after six months, a significantly improved endothelial function is reached [88]. Furthermore, periodontal treatment has been shown to reduce atherosclerotic biomarkers (e.g., IL-6, TNF-α) of individuals with cardiovascular disease and/or diabetes [89] as well as to improve the glycemic status (Hba1c levels) of diabetic patients [90,91].

## 6. Future Considerations

Periodontal disease is multifactorial and the imbalance between tissue loss and gain can occur due to various reasons, including aggressive infection, uncontrolled chronic inflammation, weakened healing, or all of the above simultaneously. Thus, successful disease management requires an understanding of different elements of the disease at the individual level and the design of personalized treatment modalities, including immunotherapies and modulators of inflammation [92,93]. With the aid of newly developed omics technologies, these novel strategies may become available for clinicians.
